# Vegetables as a vehicle for antimicrobial resistance (vAMR): An agroecosystem exploration from the One Health perspective in India

**DOI:** 10.12688/f1000research.131679.1

**Published:** 2023-03-22

**Authors:** Pachillu Kalpana, Timo Falkenberg, Sandul Yasobant, Deepak Saxena, Christiane Schreiber

**Affiliations:** 1 Department of Pharmacy, Faculty of Mathematics and Natural Sciences, University of Bonn, Bonn, NRW, 53113, Germany; 2 One Health Graduate School, Center for Development Research (ZEF), University of Bonn, Bonn, NRW, 53113, Germany; 3 Institute for Hygiene and Public Health (IHPH), Universitätsklinikum Bonn (University Hospital Bonn), Bonn, NRW, 53127, Germany; 4 School of Epidemiology & Public Health, Datta Meghe Institute of Medical Sciences (DMIMS), Wardha, Maharastra, 442004, India; 5 Centre for One Health Education, Research & Development (COHERD), Indian Institute of Public Health Gandhinagar (IIPHG), Gandhinagar, Gujarat, 382042, India

**Keywords:** Antimicrobial resistance, antibiotic-resistant bacteria, antibiotic-resistant genes, vegetables, fresh produce, agroecosystem, One Health

## Abstract

**Introduction:** Antimicrobial resistance (AMR) has emerged as one of the leading threats to public health. AMR possesses a multidimensional challenge that has social, economic, and environmental dimensions that encompass the food production system, influencing human and animal health. The One Health approach highlights the inextricable linkage and interdependence between the health of people, animals, agriculture, and the environment. Antibiotic use in any of these One Health areas can potentially impact the health of other areas. There is a dearth of evidence on AMR from the natural environment, such as the plant-based agriculture sector. Antibiotics, antibiotic-resistant bacteria (ARB), and related AMR genes (ARGs) are assumed to present in the natural environment and disseminate resistance to fresh produce/vegetables and thus to human health upon consumption. Therefore, this study aims to investigate the role of vegetables in the spread of AMR through an agroecosystem exploration from a One Health perspective in Ahmedabad, India.

**Protocol:** The present study will be executed in Ahmedabad, located in Gujarat state in the Western part of India, by adopting a mixed-method approach. First, a systematic review will be conducted to document the prevalence of ARB and ARGs on fresh produce in South Asia. Second, agriculture farmland surveys will be used to collect the general farming practices and the data on common vegetables consumed raw by the households in Ahmedabad. Third, vegetable and soil samples will be collected from the selected agriculture farms and analyzed for the presence or absence of ARB and ARGs using standard microbiological and molecular methods.

**Discussion:** The analysis will help to understand the spread of ARB/ARGs through the agroecosystem. This is anticipated to provide an insight into the current state of ARB/ARGs contamination of fresh produce/vegetables and will assist in identifying the relevant strategies for effectively controlling and preventing the spread of AMR.

**List of abbreviations**
AMR: Antimicrobial resistance
ARB: Antibiotic resistant bacteria
ARGs: AMR genes
WHO: World Health Organization
AMC: Ahmedabad Municipality Corporation
AUDA: Ahmedabad Urban Development Authority
E. coli: Escherichia coli
XLD Agar: Xylose Lysine Deoxycholate
AST: Antimicrobial Susceptibility Test
CLSI: Clinical Laboratory Standards Institute
qPCR: Quantitative Polymerase Chain Reaction
LOQ: Limit of Quantification

## Introduction

The worldwide emergence of antimicrobial resistance (AMR), altering lifesaving antibiotics, is a significant public health challenge. ^1^ The estimation exhibited that AMR is accountable for 700,000 mortalities annually worldwide and as per the current rate of resistance-related deaths, it is projected to rise to about 10 million mortalities annually by 2050. ^2^ The impact of AMR is not confined to human mortalities but is also extended to related economic losses, estimated at around $100-210 trillion in healthcare-related expenses. ^3^ A dearth of reliable data and evidence from low-middle income countries such as India contributes to underestimating the rate of AMR and related threats. Conversely, the recent Global Antimicrobial Resistance and Use Surveillance System (GLASS)-AMR 2021 report consisting of data from the national surveillance has reported the average increased burden of AMR with a significant focus on human clinical settings in India. ^4^

The major contributing factors to the AMR issue are not limited to the overuse and increased misuse of antibiotics (prescription and consumption) in human settings. ^5^ The prophylactic use and practice of sub-lethal doses of antibiotics in livestock as growth promoters to maintain health and productivity contribute to a significant part. ^6^ Most antibiotics used in human and animal sectors are not entirely metabolized in the bodies; a high percentage of administered drugs are discharged into water and soil through municipal wastewater, animal manure, sewage sludge, and biosolids. ^7^ The relationship between the consumption of antibiotics is directly associated with the emergence and promotion of antibiotic resistance in the microbial population of multiple sectors, including humans, animals, and the environment. ^8^

The environmental dimension of AMR has received comparatively less focus than AMR in human or animal health. Directly connecting antibiotics, animal production, and the environment is the practice of recycling animal waste as manure for reintroducing organic materials and nutrients to the soil to improve crop productivity. ^9^ At the same time, it presents a major pathway for the dissemination of antibiotic residues, antibiotic-resistant bacteria (ARB), and AMR genes (ARGs). ^10^ Thus, agricultural sources are considered a critical source and dissemination route for AMR. ^11^ The spread of antibiotic resistance in the agricultural ecosystem is a major concern because agriculture is closely related to food safety and human health. The exposure of humans to foodborne bacterial pathogens, including ARB, through the food chain, has been reported in recent decades in foodborne outbreaks. ^12^

The enrichment of ARB and ARGs in agricultural soil, even without the apparent application of antibiotics via human agriculture practices, has raised concerns because soil ARB and ARGs can be disseminated to plants, leading to the potential spread from farms to consumers. ^13^ There are many potential pathways by which soil-associated ARB and ARGs can transfer to plant microbiomes contributing to the emergence and spread of plant resistome to the human food chain. ^14^^,^^15^ Food consumption represents a major route for human exposure to ARGs in environmental microbiomes. ^16^ Some studies indicate that the consumption of raw fruit and vegetable is a potential pathway for disseminating ARGs to human microbiomes. ^17^ It has been suggested that the long-term use of organic fertilizers causes ARGs enrichment not only of soils but also of plant phyllosphere microbiomes. ^14^ There are many potential pathways by which soil-associated ARB and ARGs can be transferred to plant microbiomes contributing to the emergence and spread of plant resistome to the human food chain. Fresh produce can be contaminated with bacterial pathogens at multiple points throughout its production by direct contact with fecal waste during farming, wastewater irrigation, use of biosolids or animal manure as fertilizer. ^11^^,^^18^ While these potential contamination pathways have been studied well for traditional pathogens, their relative contributions to the contamination of fresh produce with ARB and ARGs have not been quantified. This is essential to capture the unexplored factors contributing towards the issue of AMR to hinder the spread. Therefore, this study findings will contribute towards the data from the foods of plant origin which connects with a link of dietary exposure to humans.

The study aim is to investigate the presence of antibiotic-resistant bacteria and the distribution of antibiotic-resistant genes across the vegetable and soil at the farm level in alignment with a one health approach in Ahmedabad city, India to provide an evidence about resistance spread for future intervention strategies.

## Protocol

### Ethical statement

Ethics approval has been obtained from the Research Ethics Committee, Center for Development Research (ZEF), University of Bonn, Germany (17c_22), and the Institutional Ethics Committee of the Indian Institute of Public Health Gandhinagar, India (TRC/2022-23/10). All details collected in the study will be kept confidential and complies with the Declaration of Helsinki. Written informed consent will be taken from each participant. All methods will be carried out in accordance with relevant guidelines and regulations. ^33^

### Aim

This study aims to provide insights into the knowledge gap and investigate the role of vegetables in spread of antimicrobial resistance through an agroecosystem exploration from a One Health perspective in one of the western communities of India. More specifically, it aims to investigate the presence of antibiotic resistant bacteria and the distribution of antibiotic resistant genes across the soil-vegetable continuum at the farm level with a One Health approach in Ahmedabad city, India.

The specific research questions are:

1. Which antibiotic resistant bacteria (ARB) and antibiotic resistant genes (ARGs) are already published or known to (or can be supposed to) be widespread in soil-plant-fruit continuum of the South Asia region?
2. What is the risk of AMR spread across soil-plant-fruit continuum in agriculture farms of Ahmedabad, India?
  2.1. How can the general agriculture practices of farmers in the region of Ahmedabad, India be characterized and which potential risk factors can be identified?
  2.2. Can ARB and/or ARGs be detected in the fresh produce/vegetables that are consumed raw or/and in the soil where these plants grow in the region of Ahmedabad, India?

### Study design

This present study is an interdisciplinary social-ecological study with the prime aim to investigate and characterize the antimicrobial resistance in vegetables and soil on harvest, which network in an intimate proximity and their possible transmission pathway along the environment-human direction using a One Health approach. A mixed-method approach is adopted: secondary data analysis (review) and primary data collection including a survey of the general agricultural practices and laboratory analysis (microbiological and molecular), which will be merged in a triangulation concept to converge the findings and generate outcomes related to the possibility of vegetables as vehicles for AMR.

### Study settings

**Figure 1.**
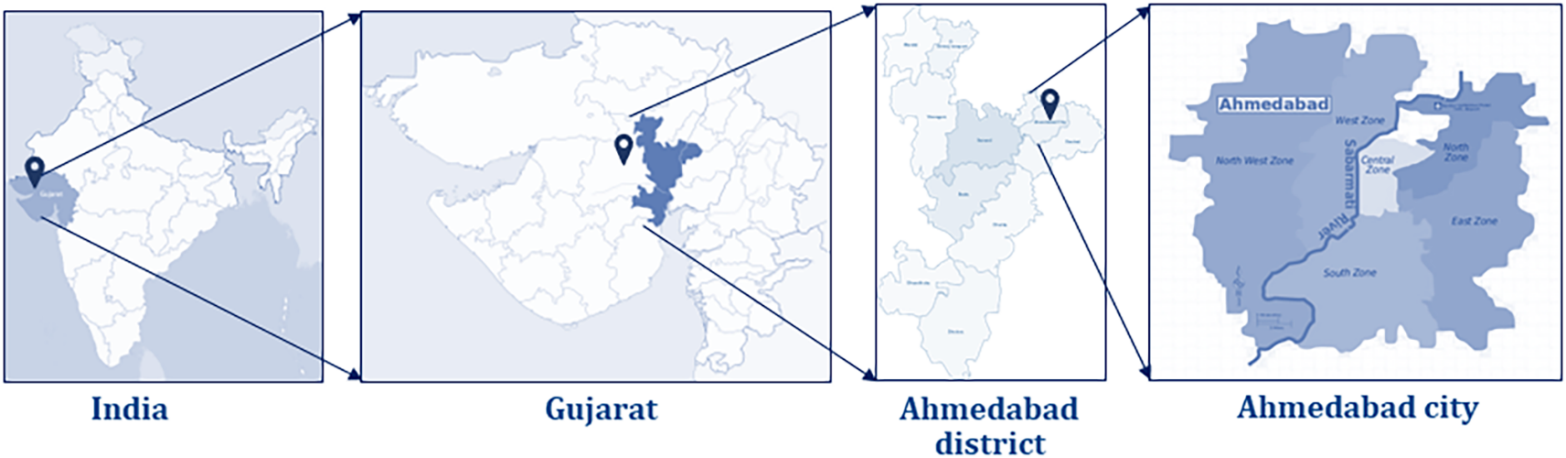
vAMR Project study site indicating the Ahmedabad city in Gujarat, India.

The proposed study will be conducted in one of the largest and most populous cities of the Western state, Gujarat, India, known as Ahmedabad ( Figure 1). Ahmedabad is located in the central part of Gujarat on the banks of the Sabarmati river, with a population of 5,633,927. ^19^ Ahmedabad's population has a combination of both vegetarian (60%) and non-vegetarian (40%) consumption patterns. ^20^ Gujarat state has an outstanding contribution to the country’s horticulture sector. ^21^

Ahmedabad city is located in the north-central part of the Ahmedabad district in Gujarat. Ahmedabad municipality was established in 1873 and later upgraded to Ahmedabad Municipality Corporation (AMC), divided into six municipal zones (north, south, east, central, west and new west) consisting of a total of 64 wards. Ahmedabad Urban Development Authority (AUDA) was established on 1st February 1978 with the prime objective to carry out the sustained, planned development of the area falling outside the periphery of Ahmedabad Municipal Corporation. The AUDA encompasses an area of 186,600 hectares, including Ahmedabad City (Municipal Corporation of 44,950 Hectares). The AUDA developed a comprehensive development plan for Ahmedabad city and the district, including the current general agriculture zone and the prime agriculture zone. The general agricultural zone is intended to provide areas with large parcels that are conducive to growing crops and the keeping of farm animals and poultry for the production of milk, wool, eggs, and other farm products. This zone is also intended to permit limited commercial and light industrial activities that support agriculture or are connected to the agricultural industry. The prime agriculture zones represent large, generally contiguous blocks of land that enable current and future opportunities for agriculture.

The increasing urbanization of Ahmedabad city observably led to developing more residential areas in the central part, shifting the agriculture practitioners involved in both livestock and plant production to the outskirts of the urban and peri-urban areas of the city. A large part of the general agriculture zones of the city is located adjacent to the city's sewage treatment plant. The general agriculture zone in the east of the city is known as a hotspot for livestock farmers. This area produces a significant amount of animal waste, the primary fertilizer source of the farms in this agriculture zone. The agriculture zone on the city's west side is located far from the sewage treatment plant and only small-scale livestock farming is being practiced.

This study is planned to be implemented in the general agriculture zones of Ahmedabad city because of the established and in-process use of land for agriculture purposes.

### Research design

The study starts with a large-scale literature-based analysis of the information on the qualitative resistance data, abundances, and patterns on ARB and ARGs on fresh produce and food production environment in context of South Asia (Research question 1). The field exploration starts with recruitment of farms, followed by a survey of the general agricultural practices in the study area and for the identification of vegetables that are produced by the farmers and also consumed raw, to identify potential risk factors for the spread of resistances (objective 2.1). The sampling of the raw consumed vegetables and the agricultural soil will be performed at the farm-level. The sampled vegetables and soil will then be microbiologically analyzed for the presence and quantity of ARB and ARGs. The gained information on the current state of ARB and ARGs on fresh produce and the agricultural soil will be reported from Ahmedabad, India (objective 2.2). Further, the findings of all the three objectives will result in the identification of possible factors that may play a role in the spread of AMR through plant-based food to human due to consumption of ARB/ARGs contaminated vegetables. Recommendations of intervention strategies will be developed to support involved actors and decision-makers of Ahmedabad, India.

**Methods for research question 1**

To understand the AMR background in the South Asia region in general, a systematic review methodology will be used. This systematic review aims to screen, assemble, summarize and evaluate the peer-reviewed and published evidence on the transmission of antibiotic resistances from differently fertilized soil to plants in the South Asia context, and to assess the evidence for possible human health related risks associated with consumption of antibiotic resistance contaminated vegetables produced on such fertilized soils.

The search strategy ( Table 1) is designed to identify published studies on ARB, ARGs in the soil, plants, and plants parts produced on manure amended soil and associated human health related issues. The interest is in identifying studies which looked upon the ARB and ARGs contamination of plants and plant produce (i.e., vegetables) through any pathway of spread from soil to plants. The identification includes both lab-based and field-based studies. The systematic literature review will be conducted using two scientific online databases: PubMed and Web of Science. The time limitation will be set to the last 10 years so that the studies selected are up-to-date and to represent knowledge that is scientifically state-of-the-art, or allow identification of recent trends in resistance spread, respectively. The key search terms are generated to encapsulate the four main concepts and therefore the review pertains to: Antibiotic resistance; bacteria and genes; fresh produce/vegetables/plants/fruits; health effects; South Asia. The table below shows the terms and how they are combined.

**Table 1.** Search terms for systematic review to be undertaken under the vAMR project, India.

Antimicrobial Resistance- related terms	Agriculture and fresh produce-related terms	Location
antimicrobial resistan$, antibiotic-resistan$ bacteria, antibiotic resistan$ genes, antimicrobial- resistant$ organisms, antibiotic -resistant pathogen$	agriculture$, farming$, fresh agriculture produc$, fresh produc$, fresh vegetable$, vegetable$, raw vegetable$, salad vegetable$, leafy green$, salad$, fruit$	South Asia$, Afghanistan$, Bangladesh$, Bhutan$, India$, Maldives$, Nepal$, Pakistan$, Sri Lanka$

### Criteria for inclusion and exclusion

The published peer-reviewed literature that documents information regarding the ARB and/or ARGs presence in plants, plant produce, vegetables, and the plant-fruit-soil continuum will be included in the review. Studies documenting the impact of plants and plant produce containing ARB and/or ARGs on human health will also be included. The review will be restricted to studies that explicitly explored the South Asia region's ARB and ARGs plant and soil environment context. The geographical restriction will involve the field site of the study conducted in South Asia, irrespective of the authors' affiliations. There will be no restriction on the design of the study. Studies published in languages other than English will be excluded. The outcomes targeted by this review will include the incidence of ARB and ARGs on plants and plant produce, from the soil environment, and the possible related human health risks due to consumption of such plant produce to define/plan next research steps more precisely.

### Data extractions

Data will be extracted from the eligible studies to summarize and tabulate important findings. Data will be extracted and tabulated about the type and prevalence of ARB/ARGs, type of food source, study design, location of the study, methodological approach, key findings. The results form an important evidence base for the selection of ARB and ARGs for the next objective of the study.

**Method for research question 2**

### Objective 2.1

The AMR field investigation is intended to capture a baseline for the further exploration of trends in vegetables and the soil used for the production of vegetables. It includes selective bacteria cultivation and identification, antibiotic resistance (pattern), and resistance genes. According to Masterton (2008), the basic protocol includes microbiological culturing and a validated antimicrobial susceptibility testing method for the first step. ^22^ The basic protocol includes methods to gain better insights into the collection of ARGs that may circulate in the environment, leading to new bacterial serotype-resistance gene combinations and can be transmitted to humans through food consumption. ^23^ The molecular methodology thus will be followed with the basic protocol to quantify the selected ARGs.

*Sampling procedure for agriculture farms*

The agriculture farm areas for this study will be selected from the general agriculture zones of Ahmedabad city. A preliminary screening will be performed in Ahmedabad city to identify the main areas involved in agricultural practices located in the urban part of the general agriculture zones, i.e., within the city’s six municipal zones to be included in this study. This screening is important given the unavailability of a database of agricultural farms in Ahmedabad city. Snowball sampling will be used to recruit the farms engaged with agriculture production. The researchers will visit the farmers physically for recruitment process in the study. Farmers will be informed about the purpose of the study and written informed consent will be obtained from the farmers. The farmers will also be informed about their right to withdraw consent at any point of time during the study period. Consent will be confirmed again before initiating the interviews and surveys. Two copies of the consent form will be prepared along with the signatures or thumb impression of farmer. One copy will remain with the farmer and one with the researcher. The farmer and farm households will be asked to list six nearby farms engaged in agriculture production activity. This process will be repeated until the desired sample size is reached. About 300 odd farms based on the type of agricultural product is aimed to be recruited from different zones of the city. The second screening of farms will be done with the principal inclusion criteria for the recruitment as vegetable production. Thus, depending on the number of farms engaged in agricultural production in a given locality, the equal number of representative farms from the different zones of the city will be surveyed and enrolled in the study. The enrolled 300 farms which will be surveyed for the general agricultural practices will then be assessed for the vegetable producing farms. 150 vegetable producing farms from the total vegetable producing farms will be selected from the different zones of the city based on their vegetable production for the next investigation step.

*Source of data and data collection methods*

A set of questions will be asked to each 300 farms engaged in agriculture production. The structured questionnaire for interviewing the farmers will be divided into five main sections: demographic characteristics, general farming practices, agriculture inputs, irrigation water and post-harvest practice. The questionnaire will also have aspects on information on the different types of crops as well as vegetables that are cultivated and those that are consumed raw by the household. This cross-sectional survey aims to identify the vegetable producing farms and the five major vegetables produced by the farms and also consumed raw by the households. The field observation will be used to validate the general farming system, fertilizer and pesticide use along with the questionnaire during the farm visits. This survey will be focused in collecting and documenting data on the general agriculture practices of farmers to explore factors, such as the use of fertilizers, pesticides, and irrigation water in farming system.

*Selection of bacteria for microbiological analysis*

The selection of bacteria is based on the recommendation of the World Health Organization (WHO). ^23^ The guidance mentions that the food of plant origin commonly intended to be consumed by humans, either fresh, minimally processed, or consumed after cooking, in that the consideration should be given to the pathogens of importance to human health and where AMR may be a hazard. The list of bacteria consists as below:

a. *Escherichia coli (E. coli)* as a measure of hygienic production and faecal indicator in general
b. *Salmonella* species as a pathogenic representative of human- and animal-derived contamination
c. *Pseudomonas* species as a common environmental bacterial contaminant serving as a sentinel for AMR

In addition to these bacteria recommended for screening by the WHO, selected species of other important bacteria can further be included in the study according to the initial systematic review of this study.

*Selection of AMR genes (ARGs) for molecular analysis*

The choice of ARGs to be included in this study will be based on the systematic review conducted for research question 1. At least two ARGs will be selected based on the most found ARGs in the context of vegetables through the systematic review. This process is important as it will provide an overview of the major ARGs which are circulating in the selected geographical area and thus comparing the presence of these genes also in Ahmedabad, India.

*Sampling procedure for microbiological analysis*

The samples for resistance investigations will be biological samples for microbiological and molecular analysis. Sampling will include soil and vegetable samples grown on the selected agricultural farms as they are considered significant vehicles for the spread of AMR. Samples will be collected when vegetables reach the marketing stage. The farm households' top five vegetables produced and consumed raw will be identified through the first survey. Five vegetable samples and the consecutive soil sample in duplicate will be collected from each selected farm. A simple random sampling method will be used for collecting vegetable samples from each farm.

### Sample collection

a) **Vegetable sampling:** The vegetable samples will be collected when they reach their commercial size through simple random sampling from each selected farm. A total of one vegetable sample in duplicates will be collected from each selected farm, thus it will be two vegetable samples from each farm. All the vegetable samples will be collected aseptically by utilizing a sterile zipper bag. After collecting samples, they will be transported to the laboratory within two hours in an icebox. ^24^
b) **Soil sampling:** There will be only one soil sample collection from each selected farm. The one soil sample in duplicates will be collected for the corresponding above selected vegetable sampling. The soil samples will be collected near the roots of the plants at a depth of 10 cm. The sampling will be taken out by utilizing a sterile shovel, and the collection of the sample will be in a sterile falcon tube. ^25^

**Sample processing:**

a) **Vegetable sample:** Vegetable samples collected from each farm will be analysed for selected bacterial contamination. The farms' samples will be first freed from any adhering soil particles. The samples will then be processed following safe handling procedures recommended for human consumption purposes (e.g., handled after hand washing, trimmed of spoiled parts, and washed thoroughly under sterile water). Vegetable samples will be chopped into small pieces with a sterile knife. 25 g of each sample will be added to 225 ml of 0.1% peptone water and will then be shaken using a horizontal shaker for 30 min at 200 rpm to get a homogenous suspension, equal to an initial dilution of 10 ^0^. Finally, a dilution series ranging from 10 ^-1^-10 ^-10^ will be prepared from this initial dilution for further processing. ^26^
b) **Soil sample:** The soil samples collected in the falcon tubes will be processed for further investigation. 10 g of soil will be taken from the collected soil and mixed with 90 ml of 0.1% peptone water in a beaker. ^26^ The mixture will be mixed well using a horizontal shaker for 30 min at 200 rpm to have the initial dilution for processing. The 10-fold serial dilution will be prepared from the initial dilution to have the dilution ranging from 10 ^-1^-10 ^-10^ for identification and enumeration of selected bacteria.

### Identification and enumeration of bacteria

***E. coli*:** A selective HiCrome ^TM^ *E. coli* Agar (Himedia, India) ^27^ will be used for cultivation of *E. coli.* The dilution range of 10 ^-1^ – 10 ^-10^ will be prepared and five consecutive dilutions of 100 μl will be spread on the agar plates. The plates will be incubated at 44°C for 24 hours. After incubation, *E. coli* will grow as blue-green colonies, and the results of the total bacterial count will be expressed as a colony-forming unit (CFU)/g of sample. The colonies will be subjected to biochemical confirmation, including an Indole production test to confirm the positive or negative isolates. The positive results for indole production isolates will be confirmed as *E. coli.* ^28^

***Salmonella* species:** A similar procedure as for *E. coli* will be carried out to identify and enumerate *Salmonella* species. Briefly, each 100 μl from the dilution range of 10 ^-1^ – 10 ^-10^will be spread on the selective media Xylose Lysine Deoxycholate (XLD) Agar. The plates will be incubated at 37°C for 24 h. After incubation, the bacterial colonies will appear pink to red-colored with or without centers. The typical colonies will be further subjected to an array of biochemical tests such as oxidase, indole production, and catalase for further confirmation ^26^

***Pseudomonas* species:** A selective and differential medium, Cetrimide Agar (Himedia, India), will be prepared to identify and enumerate *Pseudomonas* spp. Each 100 μl from the dilution range 10 ^-1^ – 10 ^-10^ will be spread on the selective media Cetrimide agar. ^29^ The plates will be incubated at 42°C for 48h. After incubation, the presumptive colonies will be identified further by a classical biochemical method, including triple sugar iron and oxidase tests. ^29^

### Antimicrobial susceptibility testing

The antimicrobial resistance pattern of the bacteria isolated from the vegetable and soil samples will be performed by antimicrobial susceptibility testing (AST). The determination of antimicrobial susceptibility will be performed by Kirby-Bauer disk diffusion technique using Muller Hinton Agar. ^30^ To check the sensitivity of the drugs, different panel of antibiotic-impregnated discs recommended for the bacterial isolates will be used as per the Clinical and Laboratory Standards Institute (CLSI) guidelines. ^31^

### Procedure for antimicrobial susceptibility testing

For pure subculture isolation, two bacterial colonies will be taken from each the vegetable and soil sample plates. The isolated colonies will be separately mixed well under aseptic conditions in physiological saline and dissolved to get a homogenous mixture comparable to 0.5 McFarland standard. The diluted bacterial suspension will then be transferred to Muller-Hinton agar plate using sterile cotton swab and the plates will be seeded uniformly rubbing the swab against the entire agar surface. After the inoculums get dried, antibiotic impregnated disks will be applied aseptically to the surface of the inoculated plates at an appropriate distance using sterile forceps. The plates will then be incubated at 37°C for 24h. ^32^ The same procedure will be followed for all the bacterial isolates included in this study: *E. coli*, *Salmonella* species and *Pseudomonas* species.

### Analysis for antimicrobial susceptibility testing

The analysis of the antibiotic susceptibility tests will be determined by the zone of inhibition. The inhibition zone will be measured for the different antibiotics used as per the bacterial isolates. The interpretation of susceptible, intermediate and resistant categories will be assigned on the basis of the critical points recommended by the CLSI guidelines. ^31^

### Sample processing for molecular analysis

I. **DNA extraction:**

a) **Soil sample:** The soil sample collected will be processed for DNA extraction. The HiPurA ^TM^ Soil DNA purification kit will be used for the isolation of DNA from soil samples. The protocol provided with the kit will be followed for isolation of complete DNA from the soil samples. The extracted DNA will be stored in elution buffer. The eluate containing the pure soil DNA will be stored at 2-8°C if processed within short-term (24-48 hours), otherwise for long-term duration -20°C or lower temperature (-80°C) is recommended by the manufacturer.
b) **Plant sample:** The plant parts sample collected will be processed for DNA extraction. The isolation of plant genomic DNA will be processed using HiPer® Plant Genomic DNA Extraction kit. The whole plant genomic DNA will be extracted following the protocol provided with the kit. The eluate containing pure extracted genomic DNA will be stored for short term storage (24-48 h) at 2-8°C as recommended. For long-term storage, -20°C or lower temperature (-80°C) is recommended. The Elution Buffer will help to stabilize the DNA at these temperatures.

II. **Detection and quantification of ARGs**

 The extracted DNA of both, soil and plant samples, will be analyzed for the presence and the quantification of ARGs. The standardized protocol for the detection and quantification after standardization will be performed by Quantitative Polymerase Chain Reaction (qPCR). Melting curves will obtained to confirm the amplification specificity. The qPCR reactions will be performed by triplicates, using a fixed dilution of raw DNA extract.

### Molecular analysis

The quantification standards will be obtained using TaqMan ^TM^ Comprehensive Microbiota control. Standard curves (Ct per log copy number) for quantification of all genes will be obtained for each run. Gene copy number of standards will be determined via the genomic DNA concentration, applying the DNA copy number and the Dilution calculator algorithms. Three technical replicates will be run for the standard curves; complete reaction mixes plus nuclease free water, but without DNA sample were used as a negative control for qPCR reactions. ARGs concentration in samples will be calculated using the standard curve equation and the measure Ct value. The quality control of raw Ct values for standard curve and unknown samples will be performed before the analysis. The limit of quantification (LOQ) will be defined as the lowest point on the linear part of the standard curve: 100 gene copies per reaction for ARGs for 16S rDNA. Copy numbers of all ARGs will be normalized to gram of sample (gene copies g ^−1^).

### Availability of data and materials

Data from this study will be available at the Center for Development Research (ZEF), Bonn, Germany, after the completion of this study. Researchers who meet the criteria for access to confidential data are encouraged to approach Ms. Ana Maria, Coordinator Fortschrittskolleg ‘One Health’, Center for Development Research (ZEF), Bonn, Genscherallee 3, 53113 Bonn, Germany. Email: health@uni-bonn.de

## Conclusion

The benefit and expected outcome of this study will be the collection of baseline data for the AMR in the fresh produce and soil in the Ahmedabad, India. This will be the first of its kind study bringing exploration of the agroecosystem and One Health approach together in India AMR loads in vegetables and corresponding soil will be linked to the agricultural practices followed by the farmers. With the objectives of this study, it will not only develop evidence on the possible routes of AMR spread and risk factors, but also will facilitate recommended WHO AMR surveillance in the plant-based agriculture through One Health approach. This vAMR study targets two broad level of dissemination. First the results will be published in the scientific journals as part of the evidence contribution from India. Second the key findings will be disseminated through the regional workshops with the policy makers, program managers, and other relevant stakeholders engaged in the concerned sectors. Recommendations from this study could be a potential source for the future surveillance of AMR for benchmarking its status, developing intervention strategies and prioritizing effective evidence-based actions. This will also provide the evidence for including the different reservoirs of environment as one of the major AMR surveillance point in the Indian National Action Plan for AMR surveillance.

## Data Availability

No data are associated with this article. Repository: PRISMA-P checklist for ‘Vegetables as vehicle for antimicrobial resistance (vAMR): An agroecosystem exploration from the One Health perspective in India’. https://doi.org/10.6084/m9.figshare.22188334. ^33^ Data are available under the terms of the Creative Commons Zero “No rights reserved” data waiver (CC0 1.0 Public domain dedication).

## References

[1] Robinson TP Bu DP Carrique-Mas J : Antibiotic resistance is the quintessential One Health issue. *Trans. R. Soc. Trop. Med. Hyg.* 2016;110:377–380. 10.1093/trstmh/trw048 27475987 PMC4975175

[2] Pokharel S Raut S Adhikari B : Tackling antimicrobial resistance in low-income and middle-income countries. *BMJ Glob. Health.* 2019;4:e002104. 10.1136/bmjgh-2019-002104 31799007 PMC6861125

[3] Antimicrobial Resistance: Tackling a crisis for the health and wealth of nations.

[4] GLASS-AMR: Global Antimicrobial Resistance and Use Surveillance System (GLASS) Report: 2021. 2021. Accessed 11 Nov 2021. Reference Source

[5] Ayukekbong JA Ntemgwa M Atabe AN : The threat of antimicrobial resistance in developing countries: causes and control strategies. Antimicrob Resist. *Infect. Control.* 2017;6:6. 10.1186/s13756-017-0208-x PMC5433038 28515903

[6] Graham DW Bergeron G Bourassa MW : Complexities in understanding antimicrobial resistance across domesticated animal, human, and environmental systems. *Ann. N. Y. Acad. Sci.* 2019;1441:17–30. 10.1111/nyas.14036 30924539 PMC6850694

[7] Skandalis N Maeusli M Papafotis D : Environmental Spread of Antibiotic Resistance. *Antibiotics.* 2021;10:640. 10.3390/antibiotics10060640 34071771 PMC8226744

[8] Cristino JM : Correlation between consumption of antimicrobials in humans and development of resistance in bacteria. *Int. J. Antimicrob. Agents.* 1999;12:199–202. 10.1016/S0924-8579(99)00052-7 10461837

[9] Hoover NL Law JY Long LAM : Long-term impact of poultry manure on crop yield, soil and water quality, and crop revenue. *J. Environ. Manag.* 2019;252:109582. 10.1016/j.jenvman.2019.109582 31614262

[10] Tien YC Li B Zhang T : Impact of dairy manure pre-application treatment on manure composition, soil dynamics of antibiotic resistance genes, and abundance of antibiotic-resistance genes on vegetables at harvest. *Sci. Total Environ.* 2017;581-582:32–39. 10.1016/j.scitotenv.2016.12.138 28076772

[11] He Y Yuan Q Mathieu J : Antibiotic resistance genes from livestock waste: occurrence, dissemination, and treatment. *npj Clean Water.* 2020;3:1–11. 10.1038/s41545-020-0051-0

[12] Zhang L Kinkelaar D Huang Y : Acquired antibiotic resistance: Are we born with it? *Appl. Environ. Microbiol.* 2011;77:7134–7141. 10.1128/AEM.05087-11 21821748 PMC3194877

[13] Ondon BS Li S Zhou Q : Sources of Antibiotic Resistant Bacteria (ARB) and Antibiotic Resistance Genes (ARGs) in the Soil: A Review of the Spreading Mechanism and Human Health Risks. *Rev. Environ. Contam. Toxicol.* 2021;256:121–153. 10.1007/398_2020_60 33948742

[14] Chen QL Cui HL Su JQ : Antibiotic Resistomes in Plant Microbiomes. *Trends Plant Sci.* 2019;24:530–541. 10.1016/j.tplants.2019.02.010 30890301

[15] Zhang YJ Hu HW Chen QL : Transfer of antibiotic resistance from manure-amended soils to vegetable microbiomes. *Environ. Int.* 2019;130:104912. 10.1016/j.envint.2019.104912 31220751

[16] Bhunia AK Liu Y Kniel K : Characteristics and Global Occurrence of Human Pathogens Harboring Antimicrobial Resistance in Food Crops: A Scoping Review. 2022;6:824714.

[17] Rahman M Alam M-U Luies SK : Contamination of Fresh Produce with Antibiotic-Resistant Bacteria and Associated Risks to Human Health: A Scoping Review. *Int. J. Environ. Res. Public Health.* 2021;19:360. 10.3390/ijerph19010360 35010620 PMC8744955

[18] Alegbeleye OO Singleton I Sant’Ana AS : Sources and contamination routes of microbial pathogens to fresh produce during field cultivation: A review. *Food Microbiol.* 2018;73:177–208. 10.1016/j.fm.2018.01.003 29526204 PMC7127387

[19] Ministry of Home Affairs: Census of India Website: Office of the Registrar General & Census Commissioner, India. 2011. Accessed 25 Nov 2021. Reference Source

[20] Times of India: “Veg” Gujarat has 40% non-vegetarians|Ahmedabad News - Times of India. 2016. Accessed 25 Nov 2021. Reference Source

[21] Gujarat Livelihood Promotion: Horticulture - Sector Overview|Sector wise Development|Activities|Gujarat Livelihood Promotion Company. 2011. Accessed 25 Nov 2021. Reference Source

[22] Masterton R : The importance and future of antimicrobial surveillance studies. *Clin. Infect. Dis.* 2008;47:S21–S31. 10.1086/590063 18713046

[23] WHO: Joint FAO/WHO expert meeting in collaboration with OIE on foodborne antimicrobial resistance: role of the environment, crops and biocides: meeting report. 2018. Accessed 17 Nov 2021. Reference Source

[24] Schwaiger K Helmke K Hölzel CS : Antibiotic resistance in bacteria isolated from vegetables with regards to the marketing stage (farm vs. supermarket). *Int. J. Food Microbiol.* 2011;148:191–196. 10.1016/j.ijfoodmicro.2011.06.001 21700353

[25] Cerqueira F Matamoros V Bayona JM : Antibiotic resistance gene distribution in agricultural fields and crops. A soil-to-food analysis. *Environ. Res.* 2019;177:108608. 10.1016/j.envres.2019.108608 31377583

[26] Abdus Sobur M Momen Sabuj A al Sarker R : Antibiotic-resistant Escherichia coli and Salmonella spp. associated with dairy cattle and farm environment having public health significance. *Vet World.* 2019;12:984–993. 10.14202/vetworld.2019.984-993 31528022 PMC6702575

[27] Antony AC Paul MK Silvester R : Comparative evaluation of EMB agar and hicrome E. coli agar for differentiation of green metallic sheen producing non E. Coli and typical E. Coli colonies from food and environmental samples. *J. Pure Appl. Microbiol.* 2016;10:2863–2870. 10.22207/JPAM.10.4.48

[28] Kannan MN Badoni A Chamoli V : Advances in Agriculture and Natural Sciences for Sustainable Agriculture (October 12 &13, 2018) Isolation and characterization of bacterial isolates from agriculture field soil of Roorkee region. ~ 108 ~. *J. pharmacogn. phytochem.* 2018;5:108–10.

[29] Ruiz-Roldán L Rojo-Bezares B Lozano C : Occurrence of pseudomonas spp. In raw vegetables: Molecular and phenotypical analysis of their antimicrobial resistance and virulence-related traits. *Int. J. Mol. Sci.* 2021;22:12626. 10.3390/ijms222312626 34884433 PMC8657893

[30] EUCAST: Disk Diffusion Method for Antimicrobial Susceptibility Testing-Antimicrobial susceptibility testing EUCAST disk diffusion method. 2021. 10.1111/apm.70002 PMC11807598 39923774

[31] Weinstein MP Patel JB Bobenchik AM : M100 Performance Standards for Antimicrobial Susceptibility Testing A CLSI supplement for global application. *Performance Standards for Antimicrobial Susceptibility Testing Performance Standards for Antimicrobial Susceptibility Testing.* 2020.

[32] Garedew L Hagos Z Addis Z : Prevalence and antimicrobial susceptibility patterns of Salmonella isolates in association with hygienic status from butcher shops in Gondar town, Ethiopia. *Antimicrob. Resist. Infect. Control.* 2015;4:1–7. 26113974 10.1186/s13756-015-0062-7 PMC4480883

[33] Kalpana P Falkenberg T Yasobant S : PRISMA-P-checklist_vAMR_Kalpana P et al, 2023.doc.figshare. Online resource.2023. 10.6084/m9.figshare.22188334.v1

