# Supplementary material for: Agroecosystem exploration for Antimicrobial Resistance in Ahmedabad, India: A Study Protocol
Source: F1000Res. 2024 Mar 25;12:316. Originally published 2023 Mar 22. [Version 2] doi: 10.12688/f1000research.131679.2 (PMC11026950; doi:10.12688/f1000research.131679.2)
Supplement: Peer review history

# Reviewer report: Reviewer response for version 2

**DOI:** 10.5256/f1000research.161818.r258904

**Published:** 2024-04-18

**Authors:** Karin Schwaiger

**Affiliations:** 1 Unit of Food Hygiene and Technology, Institute for Food Safety, Food Technology and Veterinary Public Health, University of Veterinary Medicine, Vienna, Austria

The authors addressed all questions and comments and updated the manuscript accordingly, if applicable.

Is the study design appropriate for the research question?

Yes

Is the rationale for, and objectives of, the study clearly described?

Yes

Are sufficient details of the methods provided to allow replication by others?

Partly

Are the datasets clearly presented in a useable and accessible format?

Not applicable

Reviewer Expertise:

Microbiology, antibiotic resistance, bacteriology, molecular biology

I confirm that I have read this submission and believe that I have an appropriate level of expertise to confirm that it is of an acceptable scientific standard.

# Reviewer report: Reviewer response for version 2

**DOI:** 10.5256/f1000research.161818.r258903

**Published:** 2024-04-10

**Authors:** William Calero-Cáceres

**Affiliations:** 1 Department of Food and Biotechnology Science and Engineering, Technical University of Ambato, Ambato, Tungurahua, Ecuador

The article was improved according to the given recommendations.

Is the study design appropriate for the research question?

No

Is the rationale for, and objectives of, the study clearly described?

Partly

Are sufficient details of the methods provided to allow replication by others?

No

Are the datasets clearly presented in a useable and accessible format?

Not applicable

Reviewer Expertise:

Antibiotic resistance, bacteriophages, molecular analysis, eDNA, Salmonella, Whole-genome sequencing

I confirm that I have read this submission and believe that I have an appropriate level of expertise to confirm that it is of an acceptable scientific standard.

# Reviewer report: Reviewer response for version 1

**DOI:** 10.5256/f1000research.144543.r195948

**Published:** 2023-09-25

**Authors:** William Calero-Cáceres

**Affiliations:** 1 Department of Food and Biotechnology Science and Engineering, Technical University of Ambato, Ambato, Tungurahua, Ecuador

The manuscript by Kalpana et al. offers a compilation of methodologies intended to address two research questions: "1. Which antibiotic-resistant bacteria (ARB) and antibiotic-resistant genes (ARGs) are prevalent, or presumed to be widespread, within the soil-plant-fruit continuum in the South Asia region?" and "2. What is the risk of AMR spreading across the soil-plant-fruit continuum in agricultural farms of Ahmedabad, India?" The article is classified as a "Study Protocol"-type and is supported by F1000Research.

 However, the title "Vegetables as a vehicle for antimicrobial resistance (vAMR): An agroecosystem exploration from the One Health perspective in India" implies that conclusive research has been conducted, leading readers to expect evidence within the article supporting the notion that vegetables act as vectors of AMR determinants. I recommend modifying the title to more accurately reflect the content, emphasizing that this manuscript primarily proposes protocols for similar studies.

 The criteria for the "Selection of AMR genes (ARGs) for molecular analysis" are vague. Why only two ARGs? Most studies often include common ARGs like sul1, tetA, ermB. The rationale should be modified to emphasize clinically relevant or emerging ARGs.

 Concerning the source of data and data collection methods, the reasoning behind choosing 300 samples needs a thorough explanation.

 In the section on the identification and enumeration of bacteria, standardized methodologies like ISO or BAM should be detailed. The mentioned methodologies lack validation. For instance, direct plating is suggested for Salmonella enumeration, but ISO 6579 recommends pre-enrichment and enrichment steps. The methodological limitation for Salmonella does not allow direct quantification from the sample.

 In procedures for antimicrobial susceptibility testing, the rationale for selecting specific antibiotics should be included. As it stands, the description does not significantly contribute to current knowledge or provide guidance for conducting studies as suggested.

 Regarding molecular analysis, it is mentioned that qPCR will be applied for the quantification of specific ARGs. However, the selection rationales for these genes are missing. Please include references detailing the initial validation of the techniques you intend to develop.

 Based on my experience, this article is currently incomplete and requires more in-depth analysis to serve as a foundational resource for studies about AMR following the One Health approach. I earnestly recommend publishing your article once the protocols are described in greater detail and, ideally, results are included.

Is the study design appropriate for the research question?

No

Is the rationale for, and objectives of, the study clearly described?

Partly

Are sufficient details of the methods provided to allow replication by others?

No

Are the datasets clearly presented in a useable and accessible format?

Not applicable

Reviewer Expertise:

Antibiotic resistance, bacteriophages, molecular analysis, eDNA, Salmonella, Whole-genome sequencing

I confirm that I have read this submission and believe that I have an appropriate level of expertise to state that I do not consider it to be of an acceptable scientific standard, for reasons outlined above.

# Response

**Published:** 2024-03-14

**Authors:** Pachillu Kalpana

**Affiliations:** University of Bonn, Germany

**Comment 1: **The manuscript by Kalpana et al. offers a compilation of methodologies intended to address two research questions: "1. Which antibiotic-resistant bacteria (ARB) and antibiotic-resistant genes (ARGs) are prevalent, or presumed to be widespread, within the soil-plant-fruit continuum in the South Asia region?" and "2. What is the risk of AMR spreading across the soil-plant-fruit continuum in agricultural farms of Ahmedabad, India?" The article is classified as a "Study Protocol"-type and is supported by F1000Research.

**Response: **Thanks for your time reviewing the work and your kind reflections.

**Changes: **No change

**Comment 2: **However, the title "Vegetables as a vehicle for antimicrobial resistance (vAMR): An agroecosystem exploration from the One Health perspective in India" implies that conclusive research has been conducted, leading readers to expect evidence within the article supporting the notion that vegetables act as vectors of AMR determinants. I recommend modifying the title to more accurately reflect the content, emphasizing that this manuscript primarily proposes protocols for similar studies.

**Response: **You are totally right; thanks for the advice. The title has been changed to:

 ''Agroecosystem exploration for Antimicrobial Resistance in Ahmedabad, India: A Study Protocol.''

**Changes:** The title of the manuscript has been revised.

**Comment 3: **The criteria for the "Selection of AMR genes (ARGs) for molecular analysis" are vague. Why only two ARGs? Most studies often include common ARGs like sul1, tetA, ermB. The rationale should be modified to emphasize clinically relevant or emerging ARGs.

**Response: **Thank you for pointing this out. The sentence reflected the need to select at least two; however, if the review finds more, they will also be included in the molecular analysis process. To clear up any misunderstanding, the sentence has been rephrased. Also, with the updated investigation plan, a greater number of ARGs can also be analysed.

**Changes:** The manuscript is updated from lines 268-270 in track changes mode. 

**Comment 4: **Concerning the source of data and data collection methods, the reasoning behind choosing 300 samples needs a thorough explanation.

**Response: **Thanks for your kind reflections.

 The manuscript has been updated to clarify the idea of the samples. In the first step, 300 farms will be selected for the survey, and in the second step, 300 samples, including 150 vegetable samples and 150 soil samples, will be collected. Choosing 300 samples for the survey depends heavily on funding availability and the time frame. We have added the same limitation.

 “The limitation is because of funding and time frame available.”

**Changes: **The manuscript is updated from lines 221-222 and 226-231 in track changes mode.

**Comment 5: **In the section on the identification and enumeration of bacteria, standardized methodologies like ISO or BAM should be detailed. The mentioned methodologies lack validation. For instance, direct plating is suggested for Salmonella enumeration, but ISO 6579 recommends pre-enrichment and enrichment steps. The methodological limitation for Salmonella does not allow direct quantification from the sample.

**Response: **Thank you for your suggestion. The reason for the identification and enumeration of bacteria not according to standardized methods like ISO and BAM is that those are developed and optimized for human samples or other samples containing low background flora, such as drinking water or food. Environmental samples, e.g., soil and plants, as investigated here, or even wastewater or manure, contain much more background flora, which interferes with the culture assays up to non-analyzability, especially if target bacteria concentrations are low.

 For medical/hygienic surveillance purposes, pre-enrichment is okay, but that leads to non-quantifiable results concerning the original concentrations in a sample.

 Choosing a methodology that is known to work with environmental samples and, moreover, is adapted to them seems to be more useful for our research objective, and the intended results are more likely to be yielded. Therefore, the published articles that have reported the data on the same have been used as reference for the methodology.

**Changes: **The manuscript has been updated, and a note has been added from lines 346-357 in track changes mode.

**Comment 6: **In procedures for antimicrobial susceptibility testing, the rationale for selecting specific antibiotics should be included. As it stands, the description does not significantly contribute to current knowledge or provide guidance for conducting studies as suggested.

**Response: **You are right; we missed that information in part.

 The antibiotic panel selection is mainly based on the pre-selected bacteria. We added information about the selection of antibiotics for susceptibility testing into the chapter “Method for research question 2.”

 Concerning the testing method, we are not sure if the sentence was not clearly describing the rationale.

 We revised the text of the three sub-chapters dealing with antimicrobial susceptibility testing.

**Changes: **The manuscript is updated from lines 260-265 in track changes mode.

**Comment 7: **Regarding molecular analysis, it is mentioned that qPCR will be applied for the quantification of specific ARGs. However, the selection rationales for these genes are missing. Please include references detailing the initial validation of the techniques you intend to develop.

**Response: **The manuscript has been updated concerning molecular analysis to improve the data and answer the objectives. We switched to whole metagenomic analysis instead of qPCR because of a discussion with the labs working in the field of AMR. It has been argued that the qPCR method is more sensitive and suitable for absolute quantification of target genes, while whole metagenomics has the potential to provide an overview of the genes as well as of the respective relative abundance of the antibiotic resistance genes. This switch will help focus on the selected genes through review and also find the presence of new resistant genes.

 The rationale behind the selection of ARGs depends on the first objective, which will provide evidence-based and reported data on the presence of ARGs in Southeast Asia for better geographical comparisons.

**Changes: **The manuscript is updated from lines 416-527 in track changes mode.

**Comment 8: **Based on my experience, this article is currently incomplete and requires more in-depth analysis to serve as a foundational resource for studies about AMR following the One Health approach. I earnestly recommend publishing your article once the protocols are described in greater detail and, ideally, results are included.

**Response: **Thanks for your reflection. The idea of having a protocol paper prior to the real field data collection is to provide insight into the suggested study methods. We value your suggestion and will prepare a separate manuscript once the data collection and evaluation are completed. We hope that the recent changes made to the manuscript will now be sufficient to provide a resource for conducting studies in this field of AMR.

**Changes: **The whole manuscript has been updated.

# Reviewer report: Reviewer response for version 1

**DOI:** 10.5256/f1000research.144543.r187877

**Published:** 2023-08-11

**Authors:** Karin Schwaiger

**Affiliations:** 1 Unit of Food Hygiene and Technology, Institute for Food Safety, Food Technology and Veterinary Public Health, University of Veterinary Medicine, Vienna, Austria

The authors describe a study design to determine the extent to which vegetables are a vehicle for antimicrobial resistance in Ahmedabad, India. For this purpose, they first intend to conduct a systematic review on the occurrence of antibiotic resistant bacteria (ARB) and AR genes (ARGs) on vegetables (and in soil) in South Asia. This is followed by agriculture farmland surveys to find out, among other things. about general agricultural practices and prevailing household consumption patterns. Based on the results of the upstream research, vegetable and soil samples will be collected from selected farms and analyzed for the presence of ARB and ARGs using bacteriological and molecular biological techniques.

 Overall, the study is logically and consecutively structured and explained in an understandable way. It should be emphasized that the authors apparently already have practical experience, as they are aware of some difficulties (e.g., lack of databases of agricultural farms), but found solutions for these (e.g., snowball system). It is also very good that an ethical statement is already available. The rationale for, and objectives of, the study are clearly described – although I think the term “One Health” is somewhat overused or lofty. It is true that antibiotic-resistant bacteria may play an important role for humans along the food chain. This study aims to determine prevalences, which of course can lead to a better understanding and consequently (indirectly by implementing appropriate measures/intervention strategies) also to a risk reduction. Humans themselves or samples of them, however, are not taken into account in the investigations. In this respect, I would recommend using the term "One Health" in a somewhat less inflationary way.

 I have a few more comments regarding the wording of the methodology:

 Introduction: The authors state that “The prophylactic use and practice of sub-lethal doses of antibiotics in livestock as growth promoters to maintain health and productivity contribute to a significant part.” to the AMR issue. I would be careful with this wording: In many countries, growth promoters have long been banned. Of course, this does not mean that antibiotics can no longer be used at all in animals, but only for therapeutic purposes (not prophylactic).

 Correctly, the authors recognize that organic fertilizers (e.g., manure) may play an important role in ARB transmission. They also seem to take this important point into account in their own research (important data would be e.g. animal species, storage time, temperature, hygienization measures). When later comparing the results of the present study with those of other countries, it should be recognized that in vegetable cultivation often no organic fertilizers (only mineral fertilizers) are used for hygienic reasons (e.g. in Germany). This might then explain (besides irrigation water) differences in the prevalences of certain (AR) bacteria (especially faecal indicators such as E. coli or Salmonella).

 Methods for research question 1: The authors will conduct a systematic review. They state that data will be extracted and the results will form an evidence base for the selection of ARB and ARGs for the next objective of the study. This is basically a valid approach. However, since the review focuses on Southeast Asia and not much data is available here yet, this may miss some important ARB and ARGs. Therefore, I would suggest expanding the search in this regard by including studies from other countries as well. In any case, antibiotics important in human medicine should be considered (e.g. according to WHO/Ranking of medically important antimicrobials).

 Methods for research question 2: The AMR field investigation seems to be very well thought out. A personal visit to the farms is also very important in my experience to inform the farmers about the basic idea and to arouse interest in participating. The selection of bacteria is also sensible in principle (faecal indicators, pathogens, environmental bacteria), but I am not sure if a sufficient number of E. coli and Salmonella will be isolated to do a statistical analysis afterwards (the prevalence in other studies is quite low). However, since the authors state that they will include the results of their systematic review anyway, I assume this will not be a problem (but then, not only the occurrence of ARBs should be searched but also that of bacteria on vegetables in general).

 Selection of AMR genes: See comment in “Methods for research question 1”.

 Sampling procedure: It is not clear to me how many samples are actually to be taken: first it says "Five vegetable samples....will be collected", but then at "Vegetable sampling" it says “A total of one vegetable sample…will be collected”. How does that come about? Is it related to the 5 different vegetable types (top five)? Please clarify/correct/rephrase.

 Identification and enumeration of bacteria: Possibly, one could consider investigating the aerobic mesophilic bacterial count as well, if bacteriology is performed anyway. This is only a small additional effort (even if the soil samples probably have to be diluted more than to 10^-10) and provides information about the general hygiene status of vegetables from this region (after following safe handling procedures). E. coli: the authors state that E. coli will be confirmed by a positive indole test. Since other Enterobacteriaceae are indole positive as well, I assume that the mentioned “biochemical confirmation” is able to differentiate these species from E. coli? Salmonella: I think that for Salmonella detection in this highly heterogeneous microbiota in the sample material, pre-enrichment is mandatory. It is also important to use not only peptone water but buffered peptone water to neutralize the acid produced by other competing bacteria. Please follow established protocols.

 Antimicrobial susceptibility testing: The authors will use the disk diffusion technique. This is basically a valid method – but why not using microdilution? In my opinion, this method would be better suited for the intended high throughput. Anyhow, it should follow an established protocol. In the description of the procedure it is not quite clear if it is intended to take the two bacterial colonies directly from the selective agar plates (it is important to take the colonies from neutral agar plates incubated in consistent times and temperatures). Since a biochemical confirmation of the colonies was conducted beforehand, I think my concern is unfounded, but please clarify. It is also not clear if a total of two bacterial colonies are taken or two bacterial colonies from each duplicate.

 Molecular analysis: Plant sample: Why using a plant genomic DNA extraction kit if it is intended to investigate bacterial DNA? Of course, there might be some bacterial endophytes in the plant cells, but that is certainly not the overwhelming majority – most of the bacteria will be contaminants on the surface of the vegetables. Detection and quantification of ARGs: This part of the investigation seems a bit separated from the rest of the study. Does that mean that the isolated bacteria will not be tested for ARGs, only the sample material (soil, vegetables) itself? This can be done, but it should be kept in mind that this will limit the informative value: Since many resistance genes are found in different bacterial species and, additionally, the microbiota differ greatly among different vegetable species, the results should be interpreted very carefully (e.g., only a very small fraction of the tetracyline resistance genes will origin from E. coli, but no definite statement is possible). It is also not possible to determine multidrug resistances, and it cannot be differentiated between opportunists and pathogens (e.g. for risk estimation). Standard curve: If only the sample material itself will be investigated for the presence of ARGs: I assume that the standard curve is created in the respective DNA-free (e.g. irradiated) sample material (vegetables, soil suspensions) in order to take any matrix effects into account?

Is the study design appropriate for the research question?

Yes

Is the rationale for, and objectives of, the study clearly described?

Yes

Are sufficient details of the methods provided to allow replication by others?

Partly

Are the datasets clearly presented in a useable and accessible format?

Not applicable

Reviewer Expertise:

Microbiology, antibiotic resistance, bacteriology, molecular biology

I confirm that I have read this submission and believe that I have an appropriate level of expertise to confirm that it is of an acceptable scientific standard, however I have significant reservations, as outlined above.

# Response

**Published:** 2024-03-14

**Authors:** Pachillu Kalpana

**Affiliations:** University of Bonn, Germany

**Comment 1:** The authors describe a study design to determine the extent to which vegetables are a vehicle for antimicrobial resistance in Ahmedabad, India. For this purpose, they first intend to conduct a systematic review on the occurrence of antibiotic resistant bacteria (ARB) and AR genes (ARGs) on vegetables (and in soil) in South Asia. This is followed by agriculture farmland surveys to find out, among other things. about general agricultural practices and prevailing household consumption patterns. Based on the results of the upstream research, vegetable and soil samples will be collected from selected farms and analyzed for the presence of ARB and ARGs using bacteriological and molecular biological techniques.

**Response: **Thanks for your time and reviewing the draft in detail.

**Changes: **NA

**Comment 2: **Overall, the study is logically and consecutively structured and explained in an understandable way. It should be emphasized that the authors apparently already have practical experience, as they are aware of some difficulties (e.g., lack of databases of agricultural farms), but found solutions for these (e.g., snowball system). It is also very good that an ethical statement is already available.

 Overall, the study is logically and consecutively structured and explained in an understandable way. It should be emphasized that the authors apparently already have practical experience, as they are aware of some difficulties (e.g., lack of databases of agricultural farms), but found solutions for these (e.g., snowball system). It is also very good that an ethical statement is already available.

 The rationale for, and objectives of, the study are clearly described – although I think the term “One Health” is somewhat overused or lofty. It is true that antibiotic-resistant bacteria may play an important role for humans along the food chain. This study aims to determine prevalence, which of course can lead to a better understanding and consequently (indirectly by implementing appropriate measures/intervention strategies) also to a risk reduction. Humans themselves or samples of them, however, are not taken into account in the investigations. In this respect, I would recommend using the term "One Health" in a somewhat less inflationary way.

**Response: **Thank you very much for your kind review. Yes, as a part of the study, the base information was gathered before planning for the study and to be aware of the difficulties during the process. The ethical clearance was obtained from both institutions for conducting the study.

 Thank you. You also very well mentioned the importance of ARB and its role in humans through the food chain, which describes it well as a One Health concept. But we agree completely that if we are not able to approach all the sectors of One Health, it is better to use the term, however, keeping it less inflammatory. Taking that into account, the manuscript has been updated.

**Changes: **The whole manuscript is updated.

**Comment 3: **I have a few more comments regarding the wording of the methodology:

 Introduction: The authors state that “The prophylactic use and practice of sub-lethal doses of antibiotics in livestock as growth promoters to maintain health and productivity contribute to a significant part.” to the AMR issue. I would be careful with this wording: In many countries, growth promoters have long been banned. Of course, this does not mean that antibiotics can no longer be used at all in animals, but only for therapeutic purposes (not prophylactic).

**Response: **Thank you. I agree that many developed countries banned the use of antibiotics as growth promoters and, therefore, reframed the sentence to make it much more globally sound. There are countries such as India where, due to a lack of policies for antibiotic use in animals, prophylactic use still plays a very prominent role.

**Changes: **The manuscript is updated from lines 15-23 in track changes mode.

**Comment 4: **Correctly, the authors recognize that organic fertilizers (e.g., manure) may play an important role in ARB transmission. They also seem to take this important point into account in their own research (important data would be e.g. animal species, storage time, temperature, hygienization measures). When later comparing the results of the present study with those of other countries, it should be recognized that in vegetable cultivation often no organic fertilizers (only mineral fertilizers) are used for hygienic reasons (e.g. in Germany). This might then explain (besides irrigation water) differences in the prevalences of certain (AR) bacteria (especially faecal indicators such as E. coli or Salmonella).

**Response**: Thank you for taking this point into account. This will be a good point of view to see during analysis. During the comparison of results, this might be a good explanation point for the results.

**Changes:** No changes

**Comment 5: **Methods for research question 1: The authors will conduct a systematic review. They state that data will be extracted and the results will form an evidence base for the selection of ARB and ARGs for the next objective of the study. This is basically a valid approach. However, since the review focuses on Southeast Asia and not much data is available here yet, this may miss some important ARB and ARGs. Therefore, I would suggest expanding the search in this regard by including studies from other countries as well. In any case, antibiotics important in human medicine should be considered (e.g. according to WHO/Ranking of medically important antimicrobials).

**Response: **Yes, we agree that the scope of review in only including the published literature from Southeast Asia might lead to skip other important ARB and ARGs reported from other countries. However, one of the major reasons for only selecting these areas is to have a focus on scarcity of data from this region and concentrate attention on the need for more studies in this area. This is also decided as per the mandate of the funded project.

 Regarding the WHO suggestion, the selection of bacteria is according to the WHO guidance for the importance of their role in plant-based food and their role in spread of AMR.

**Changes:** No changes

**Comment 6: **Methods for research question 2: The AMR field investigation seems to be very well thought out. A personal visit to the farms is also very important in my experience to inform the farmers about the basic idea and to arouse interest in participating. The selection of bacteria is also sensible in principle (faecal indicators, pathogens, environmental bacteria), but I am not sure if a sufficient number of E. coli and Salmonella will be isolated to do a statistical analysis afterwards (the prevalence in other studies is quite low). However, since the authors state that they will include the results of their systematic review anyway, I assume this will not be a problem (but then, not only the occurrence of ARBs should be searched but also that of bacteria on vegetables in general).

**Response: **Thank you for appreciating the investigation plans. The review will provide us with the idea of prevalence, as you have also quite clearly mentioned, which will help us in the process. I agree with you about the importance of including the total occurrence of bacteria on vegetables, which can have added value. However, the limited time and resources available to complete the project led to the compromise of this idea, focusing on some specific bacteria with much more in focus according to the WHO guidelines.  Indeed, our final analysis method does not only include ARB because identification is done in two steps: first, cultivation of bacteria species of interest, and second, resistance tests of these isolates. That was a result of the review and practical possibilities in the labs, therefore not clear when writing this study concept.

**Changes:** No changes

**Comment 7: **Selection of AMR genes: See comment in “Methods for research question 1”.

 Sampling procedure: It is not clear to me how many samples are actually to be taken: first it says "Five vegetable samples....will be collected", but then at "Vegetable sampling" it says “A total of one vegetable sample…will be collected”. How does that come about? Is it related to the 5 different vegetable types (top five)? Please clarify/correct/rephrase.

**Response: **I hope the reply to the comment for research question 1 clarifies this one here.

 The top five vegetables will be selected after the survey. During the sample collection, one vegetable sample and its soil sample will be collected in duplicate from every selected vegetable-producing farm. The collected samples from all investigated farms together will represent the top five vegetables. The sentence has been rephrased for clarification.

**Changes: **The manuscript is updated from lines 275-286 in track changes mode.

**Comment 8:**Identification and enumeration of bacteria: Possibly, one could consider investigating the aerobic mesophilic bacterial count as well, if bacteriology is performed anyway. This is only a small additional effort (even if the soil samples probably have to be diluted more than to 10^-10) and provides information about the general hygiene status of vegetables from this region (after following safe handling procedures). E. coli: the authors state that E. coli will be confirmed by a positive indole test. Since other Enterobacteriaceae are indole positive as well, I assume that the mentioned “biochemical confirmation” is able to differentiate these species from E. coli? Salmonella: I think that for Salmonella detection in this highly heterogeneous microbiota in the sample material, pre-enrichment is mandatory. It is also important to use not only peptone water but buffered peptone water to neutralize the acid produced by other competing bacteria. Please follow established protocols.

**Response: **I hope the reply to the comment for research question 1 clarifies this one here.

 The top five vegetables will be selected after the survey. During the sample collection, one vegetable sample and its soil sample will be collected in duplicate from every selected vegetable-producing farm. The collected samples from all investigated farms together will represent the top five vegetables. The sentence has been rephrased for clarification.

 We agree that investigating the aerobic mesophilic bacterial count will be a small additional effort, but as you mentioned, soil samples will need to be diluted more. Additionally, we use selective agar for coliforms. Therefore, we have considered not only reporting E. coli but also accounting for the total coliform count for the samples, which is often an indicator of cleanliness and hygiene.

 Regarding the biochemical confirmation of E. coli with indole due to the very selective media that will be used for the isolation of E. coli. Although the manufacturer says there could be growth of *Salmonella* in very rare cases. The bacterial isolates can be differentiated with their color on the agar plates but indole positive E. coli and *Salmonella* which is an indole negative bacterium can be a confirmatory test that while proceeding further there is no contamination. However, with the consultation with labs, we considered going with IMViC tests to differentiate the Enterobacteriaceae family members further.

 The pre-enrichment process for *Salmonella* detection is well understood. The suggestion provided is well taken and the buffered peptone water will be used for the process. The BPA will be used for the non- selective enrichment process which will be followed by the very selective media for isolation of *Salmonella*.  The authors thought of following the published articles protocol due to the non-sample specific protocol for *Salmonella* detection in established protocols such as BAM during the manuscript preparation. But doing pre-enrichment, we are not able to count real *Salmonella* concentrations. The researchers of the articles which has been referenced in the manuscript have used the same media and method for the isolation of *Salmonella* from vegetable samples as we choose, and they were able to find relevant numbers of *Salmonella*. 

**Changes: **The manuscript is updated from lines 321-331 in track changes mode.

**Comment 9: **Antimicrobial susceptibility testing: The authors will use the disk diffusion technique. This is basically a valid method – but why not using microdilution? In my opinion, this method would be better suited for the intended high throughput. Anyhow, it should follow an established protocol. In the description of the procedure it is not quite clear if it is intended to take the two bacterial colonies directly from the selective agar plates (it is important to take the colonies from neutral agar plates incubated in consistent times and temperatures). Since a biochemical confirmation of the colonies was conducted beforehand, I think my concern is unfounded, but please clarify. It is also not clear if a total of two bacterial colonies are taken or two bacterial colonies from each duplicate.

**Response: **The disk diffusion method is been chosen due to its much use and less expense in manual work regarding less technical equipment as compared to the microdilution method. You can test more than one antibiotic in parallel on the same plate, whereas microdilution needs a single dilution series for every antibiotic.

 In the beginning of the study, during the conceptual phase, it was not clear how many bacterial colonies will be found during the process. The idea is to test several, but eventually not all bacterial colonies (depending on their numbers found and the workload we can afford) from both, the vegetable and the respective soil, to compare their resistance patterns. The text is revised.

**Changes: **The manuscript is updated from lines 360-363 in track changes mode.

**Comment 10: **Molecular analysis: Plant sample: Why using a plant genomic DNA extraction kit if it is intended to investigate bacterial DNA? Of course, there might be some bacterial endophytes in the plant cells, but that is certainly not the overwhelming majority – most of the bacteria will be contaminants on the surface of the vegetables. Detection and quantification of ARGs: This part of the investigation seems a bit separated from the rest of the study. Does that mean that the isolated bacteria will not be tested for ARGs, only the sample material (soil, vegetables) itself? This can be done, but it should be kept in mind that this will limit the informative value: Since many resistance genes are found in different bacterial species and, additionally, the microbiota differ greatly among different vegetable species, the results should be interpreted very carefully (e.g., only a very small fraction of the tetracyline resistance genes will origin from E. coli, but no definite statement is possible). It is also not possible to determine multidrug resistances, and it cannot be differentiated between opportunists and pathogens (e.g. for risk estimation). Standard curve: If only the sample material itself will be investigated for the presence of ARGs: I assume that the standard curve is created in the respective DNA-free (e.g. irradiated) sample material (vegetables, soil suspensions) in order to take any matrix effects into account?

**Response: **The protocol has been updated concerning the extraction kit that will ultimately be used for DNA extraction in the study. This results from a methodical harmonization with the other researchers who are working in this field, and it was previously shown that the kit worked on both types of samples selected in this study.

 The disk diffusion method will be used to test the isolated bacteria for AMR susceptibility. Gene detection of ARG within individual isolates will not be done. We know that, thus, we underestimate phenotypically expressed resistances and do not detect non-expressed multi-resistances, but we thought that ARG occurrence in the environment (soil and samples) does tell us more about the resistance spread of human-relevant antibiotics.

 ARG analysis in both the isolates and samples is out of our monetary budget and available time frame. Thus, we had to restrict and select the better analysis for our research objectives and keep in mind that this will limit the informative value, as you stated. However, the sample will be investigated by isolating the DNA from the complete sample using the whole metagenomic sequencing method. The AST patterns will provide us with information regarding the specific bacteria selected as per WHO suggestions, and the Whole metagenomic sequencing will provide us with more in-depth information of ARGs. This can also help the researchers compare the much better methodology to be followed in the near future and according to the budget. Whole metagenomic sequencing will help identify and cluster many gene fragments into groups. The resistant genes will be identified using the AMR databases and the potential connections of metagenomic results to phenotypically observed data. With the changed investigation plan, I hope the standard curve will no longer be required.

**Changes: **The manuscript is updated from lines 399-411 and 416-527  in track changes mode.

 Thank you very much for your comments and suggestions and for reviewing the manuscript. I hope we clarified them and updated the manuscript for better understanding and adaptability.
